# Low Concentrations of Vitamin C Reduce the Synthesis of Extracellular Polymers and Destabilize Bacterial Biofilms

**DOI:** 10.3389/fmicb.2017.02599

**Published:** 2017-12-22

**Authors:** Santosh Pandit, Vaishnavi Ravikumar, Alyaa M. Abdel-Haleem, Abderahmane Derouiche, V. R. S. S. Mokkapati, Carina Sihlbom, Katsuhiko Mineta, Takashi Gojobori, Xin Gao, Fredrik Westerlund, Ivan Mijakovic

**Affiliations:** ^1^Systems and Synthetic Biology Division, Department of Biology and Biological Engineering, Chalmers University of Technology, Gothenburg, Sweden; ^2^Computational Bioscience Research Center, King Abdullah University of Science and Technology, Thuwal, Saudi Arabia; ^3^Biological and Environmental Sciences and Engineering Division, King Abdullah University of Science and Technology, Thuwal, Saudi Arabia; ^4^Proteomics Core Facility, Sahlgrenska Academy, University of Gothenburg, Gothenburg, Sweden; ^5^Novo Nordisk Foundation Center for Biosustainability, Technical University of Denmark, Kongens Lyngby, Denmark

**Keywords:** biofilms, exopolymeric matrix, quantitative proteomics, *Bacillus subtilis*, vitamin C

## Abstract

Extracellular polymeric substances (EPS) produced by bacteria form a matrix supporting the complex three-dimensional architecture of biofilms. This EPS matrix is primarily composed of polysaccharides, proteins and extracellular DNA. In addition to supporting the community structure, the EPS matrix protects bacterial biofilms from the environment. Specifically, it shields the bacterial cells inside the biofilm, by preventing antimicrobial agents from getting in contact with them, thereby reducing their killing effect. New strategies for disrupting the formation of the EPS matrix can therefore lead to a more efficient use of existing antimicrobials. Here we examined the mechanism of the known effect of vitamin C (sodium ascorbate) on enhancing the activity of various antibacterial agents. Our quantitative proteomics analysis shows that non-lethal concentrations of vitamin C inhibit bacterial quorum sensing and other regulatory mechanisms underpinning biofilm development. As a result, the EPS biosynthesis in reduced, and especially the polysaccharide component of the matrix is depleted. Once the EPS content is reduced beyond a critical point, bacterial cells get fully exposed to the medium. At this stage, the cells are more susceptible to killing, either by vitamin C-induced oxidative stress as reported here, or by other antimicrobials or treatments.

## Introduction

Bacterial biofilms are culprits of various human infectious diseases, industrial corrosion and food contamination (Flemming et al., 2016). Bacteria within the biofilms synthesize a dense protective matrix composed of extracellular polymeric substances (EPS) (Branda et al., 2005). This matrix is mainly composed of polysaccharides, proteins and extracellular DNA (eDNA), whose continuous release leads to the establishment of a complex “mushroom-shaped” biofilm architecture (Branda et al., 2006; Barnes et al., 2012). Exopolysaccharides and proteins are the most abundant component of the biofilm matrix, defining its physico-chemical properties and morphology (Marvasi et al., 2010; Roy et al., 2017). Furthermore, the EPS serve as a food storage, which gets mobilized during extended nutrient depletion (Xiao et al., 2012). The structure of the EPS matrix varies considerably among bacterial strains, and its composition is influenced by the local environment and nutrient availability.

Antibiotics are widely used to eradicate bacterial biofilms when treating infections. However, their prolonged use increases the risk of developing multi-resistant strains, and disrupts the ecology of the residential microflora (Cegelski et al., 2008). Hence an increasing interest for chemo-prophylactic agents, which can affect biofilm formation, and thereby reduce the time and dose of antibiotics treatments (Xavier et al., 2005; Cegelski et al., 2008). Vitamin C, a major dietary micronutrient, has been shown to exhibit bactericidal activity against mycobacteria (Vilcheze et al., 2013). However, this killing effect seems to be confined to mycobacteria, since vitamin C did not kill other opportunistic bacterial pathogens, such as *Staphylococcus epidermidis, Staphylococcus aureus, Escherichia coli*, and *Pseudomonas aeruginosa* (Khameneh et al., 2016). Interestingly, vitamin C has been reported to enhance the effect of antibiotics vs. a broad spectrum of bacteria via a synergistic effect, but the mechanism of this synergy remains unclear (Kallio et al., 2012; Khameneh et al., 2016). Similarly, vitamin C has been shown to enhance the killing effect of a physical bactericidal agent, cold atmospheric plasma, against biofilms of *S. epidermidis*, *E. coli*, and *P. aeruginosa* (Helgadóttir et al., 2017). In this study we set out to characterize the mechanism of this non-lethal synergistic effect of vitamin C, which enhances the effect of antibiotics and physical killing agents.

We performed an initial characterization with several bacterial strains, and different doses of vitamin C. Our conclusion was that while the low doses of vitamin C are harmless to the planktonic bacteria, they effectively destabilizes biofilms. We then focused on an in-depth quantitative analysis with *Bacillus subtilis*, a model organism for biofilm development (Vlamakis et al., 2013). Our findings, based on quantification of the biofilm EPS content and cell viability, quantitative proteome analyses and genome-scale metabolic modeling point to a vitamin C-dependent inhibition of the synthesis of polysaccharides that form the biofilm matrix. This proceeds via inhibition of the quorum sensing and other regulatory mechanisms, leading to repression of specific biosynthetic operons. Once the EPS content is reduced beyond a critical point, bacterial cells become exposed, and more susceptible to killing by any external factors.

## Materials and Methods

### Bacterial Strains, Culture Media, and Reagents

*Bacillus subtilis* NCIB 3610, *E. coli* UTI89 and *P. aeruginosa* PAO1 were used in this study. LB (10 g of tryptone, 5 g of yeast extract and 5 g of NaCl per liter) or solid LB medium supplemented with 1.5% agar were used for the routine growth of all bacteria. Sodium ascorbate was purchased from Sigma–Aldrich.

### Bacterial Growth and Biofilm Formation

For growth analysis, an overnight bacterial culture was diluted 1:100 (1 × 10^7^ CFU) in LB medium with 1% glycerol for *B. subtilis*, plain LB medium for *P. aeruginosa* and *E. coli*, with varying concentrations of sodium ascorbate (neutral pH form). The diluted cultures were incubated at 37°C with continuous agitation (200 rpm) and the absorbance of the culture was measured periodically at 600 nm for 9 h with intervals of 1 h. *B. subtilis* biofilms were formed in LBGM medium (LB medium containing 1% glycerol; 1 mM MnSO_4_). 2–5 × 10^6^ CFU/mL of bacterial culture was inoculated into 5 mL of LBGM medium and incubated at 37°C for 24 h without agitation. *E. coli* and *P. aeruginosa* biofilms were formed on 24 well plates. 2–5 × 10^6^ CFU/mL of an overnight bacterial culture was inoculated into a 24 well plate containing 2 mL of LB broth and incubated for 24 h without agitation.

### *B. subtilis* Biofilm Analysis

For the biofilm analysis, 24 h old biofilms, grown in the presence of various concentrations of sodium ascorbate, were removed and sonicated at 10 W for 30 s to homogenize the biofilm. The homogenized suspension (5 mL) was used to determine the biomass, colony forming units (CFU), polysaccharides, protein and eDNA. Briefly, for the determination of biomass, the homogenized suspension was washed three times (5000 *g* for 20 min) with sterile water, lyophilized and weighed. For the determination of viability, an aliquot (100 μL) from the homogenized suspension was diluted serially and plated on LB agar plates to count colonies. Water insoluble polysaccharide was extracted from the lyophilized sample by using 1 N sodium hydroxide (300 μL/mg biomass for three times) and quantified by using a phenol-sulfuric acid assay as described previously (Pandit et al., 2011). For protein quantification, biofilms were collected and homogenized in 1 N NaOH (300 μL/mg biomass for three times). The supernatant from the homogenized suspensions were collected after centrifugation (5000 *g*, 20 min) and protein content was quantified by using the Bradford assay. For quantification of eDNA in the EPS matrix, the filtered supernatant from the homogenized suspension was used. eDNA was extracted by using a DNA extraction kit (Thermo Fisher Scientific) and the quantity was measured using a nanodrop UV-Vis spectrophotometer (NanoDrop 2000, Thermo Scientific).

### Fluorescence Microscopy Analysis

The effect of sodium ascorbate on *B. subtilis* biofilms was analyzed by simultaneous labeling of the bacterial cells and the polysaccharides in the biofilm. Briefly, 10 μg/mL of Alexa flour^®^ 633-labeled wheat germ agglutinin conjugate (absorbance/fluorescence emission maxima 632/647 nm; Molecular Probes Inc., Eugene, OR, United States) and 50 μg/mL of Concanavalin A, Tetramethylrhodamine conjugate (555/580 nm; Molecular Probes) was added to the culture medium during biofilm formation. The toxicity of these fluorescence probe toward the bacterial cells in biofilms was evaluated by comparison of viability and biomass with control samples. After 24 h, the biofilms were exposed to 2.5 μm of SYTO^®^ 9 green-fluorescent nucleic acid stain (480/500 nm; Molecular Probes) for 30 min. The stained biofilms were transferred to a glass slide and laser scanning confocal microscope imaging of the biofilms was performed using an LSM 710 NLO (Carl Zeiss) equipped with argon-ion and helium neon lasers. Three independent experiments were performed and image stacks from five sites per experiment were collected (*n* = 15). EPS biovolume was quantified from confocal stacks by COMSTAT (Heydorn et al., 2000). Biovolume is defined as the volume of the biomass (μm^3^) divided by substratum area (μm^2^). For the detection of reactive oxygen species (ROS) in biofilm cells, 24 h old *B. subtilis* biofilms grown with or without presence of vitamin C were stained with DAPI and CellRox deep ROS sensor (Life Technologies) as described previously (Durmus et al., 2013). Briefly, biofilms were stained with 5 μM of CellRox deep red stain for 30 min, washed with sterile water and counter- stained with DAPI for 20 min. The stained biofilms were visualized by fluorescence microscopy. To visualize the live/dead cells in *B. subtilis* biofilms grown with and without presence of vitamin C, biofilms were stained with 6.0 μM SYTO 9 and 30 μM propidium iodide (LIVE/DEAD BacLight bacterial viability kit L13152, Invitrogen, Molecular Probes, Inc., Eugene, OR, United States). Imaging was performed with a fluorescence microscope (Axio Imager 2. Carl Zeiss, Zena, Germany).

### Assay for Biofilm Formation of *E. coli* and *P. aeruginosa*

For the biofilm formation assay, 24 h old biofilms of *E. coli* and *P. aeruginosa* were rinsed three times with sterile water to remove the loosely adherent bacteria and dried for 30 min at room temperature. Dried biofilms were then stained with 1% crystal violet for 5 min without agitation. All the biofilms were washed at least five times with sterile water to remove the excess stain and dried for 1 h at room temperature. Absolute ethanol (1 mL) was added to the dried stained biofilm and agitated vigorously for 15 min to dissolve the stain. Optical density was measured at 600 nm.

### Proteome Analysis

All experiments for MS analysis were carried out in biological triplicates. 24 h biofilms of *B. subtilis* were collected and centrifuged to obtain a cell pellet. Cell lysis was performed by re-suspending the cell pellets in an SDS lysis buffer containing 4% SDS in 100 mM triethylammonium bicarbonate pH 8.5, 5 mM β-glycerophosphate, 5 mM sodium fluoride, 5 mM sodium orthovanadate and 10 mM ethylenediaminetetraacetic acid, along with a protease inhibitor cocktail (Roche). The cell extracts were boiled at 90°C for 10 min followed by sonication. The cell debris was removed by centrifugation at 13400 rpm for 30 min and the crude protein extracts were cleaned up by chloroform/methanol precipitation. Dried protein pellets were dissolved in denaturation buffer containing 8 M urea in 10 mM Tris-HCl pH 8.0. The protein lysate was reduced with 1 mM dithiothreitol and alkylated with 5.5 mM iodoacetamide in the dark, for 1 h each at room temperature. Proteins were then subjected to overnight digestion with an endoproteinase Trypsin (1:100, w/w; Pierce^TM^). The reaction was stopped by acidification with 10% trifluoroacetic acid and stage-tipped before injecting the samples into the mass spectrometer (Ishihama et al., 2006). Samples were analyzed on an Q Exactive mass spectrometer coupled to an Easy-nLC 1200 (both Thermo Fisher Scientific, Inc., Waltham, MA, United States). Chromatographic separation was performed using an in-house constructed pre-column (45 mm × 0.075 mm I.D) and analytical (200 mm × 0.075 mm I.D.) column set up packed with 3 μm Reprosil-Pur C18-AQ particles (Dr. Maisch GmbH, Ammerbuch, Germany). Peptides were injected onto the column with solvent A (0.2% formic acid in water) at a flow rate of 300 nL/min and 500 bars. Peptides were then eluted using a segmented gradient of 7–27% B-solvent (80% acetonitrile with 0.2% formic acid) over 45 min, 27–40% B over 5 min, 40–100% B over 5 min with a final hold at 100% B for 10 min. The mass spectrometer was operated on a data-dependent mode. Survey full-scans for the MS spectra were recorded between 400 and 1600 Thompson at a resolution of 70,000 with a target value of 1e6 charges in the Orbitrap mass analyzer. The top 10 most intense peaks from the survey scans of doubly or multiply charged precursor ions were selected for fragmentation with higher-energy collisional dissociation (HCD) with a target value of 1e5 in the Orbitrap mass analyzer in each scan cycle. Dynamic exclusion was set for 30 s. Triplicate injections (technical replicates) were carried out for each of the samples for label free quantitation (LFQ).

### Data Processing and Analysis

Acquired MS spectra were processed with the MaxQuant software suite (version 1.5.3.30) (Cox et al., 2009), integrated with an Andromeda search engine. Database search was performed against a target-decoy database of *B. subtilis* 168 downloaded from UniProt (taxonomy ID 1423), containing 4,195 protein entries, and additionally including also 248 commonly observed laboratory contaminant proteins. Endoprotease Trypsin/P was set as the protease with a maximum missed cleavage of two. Carbamidomethylation (Cys) was set as a fixed modification. Label free quantification was enabled with a minimum ratio count of two. A false discovery rate of 1% was applied at the peptide and protein level individually for filtering identifications. Initial mass tolerance was set to 20 ppm. In case of the main search, the peptide mass tolerance of precursor and the fragment ions were set to 4.5 and 20 ppm, respectively. Downstream bioinformatics analysis was performed using Perseus version 1.5.3.2 (Tyanova et al., 2016). Grouping of proteins with similar expression profiles was achieved by hierarchical clustering analysis. Log10 transformation of mean LFQ intensities of proteins was performed for all the tested conditions. Missing values were replaced from the normal distribution via imputation. Hierarchical clustering was performed on *Z*-score transformed values using Euclidean as a distance measure and Average linkage cluster analysis. Significance B (*p* ≤ 0.05) was calculated to identify significantly regulated proteins in each of the ascorbate treatment conditions relative to the control.

### Reconstruction of Context-Specific Models Using Proteomics Data

Log10 LFQ protein intensities were used to generate sodium ascorbate concentration specific models by mapping the protein intensities to the genome-scale metabolic model of *B. subtilis* (Bs-iYO844) (Oh et al., 2007). Log10 LFQ protein intensities from biological replicates (three replicates for each sodium ascorbate concentration and 2 for the control) were averaged and used to constrain the fluxes in the associated reaction using Gene Inactivity Moderated by Metabolism and Expression (GIMME) (Becker and Palsson, 2008). GIMME was run using 90% of the objective function threshold and 50th percentile of proteins expression level. Since properly constrained reactions do not demonstrate uniform distributions of feasible steady-state fluxes, the range and distribution of feasible metabolic flux for each reaction were determined by using *Markov Chain Monte Carlo* (MCMC) sampling (Lewis et al., 2012). To do this, a large number of feasible sets of metabolic fluxes were randomly moved within the solution space until they were well mixed, thereby sampling the entire solution space (Lewis et al., 2012). This sampling process yielded a distribution of feasible steady-state fluxes for each reaction. Sampling was done using *gpSampler* with default settings from the COBRA toolbox (Schellenberger et al., 2011) using Gurobi (Gurobi Optimization, Inc., Houston, TX, United States) and MATLAB^®^ (The MathWorks Inc., Natick, MA, United States). Averaged sampled predicted flux distributions for each reaction at each sodium ascorbate concentration were compared to those from the control model in order to identify reactions (and their associated genes) that have significantly altered flux rates upon adding sodium ascorbate to *B. subtilis*.

### Statistical Analysis

The data are presented as the mean ± standard deviation. Intergroup differences were estimated by one-way analysis of variance (ANOVA), followed by a *post hoc* multiple comparison (Tukey) test to compare the multiple means. Differences between values were considered to be statistically significant when the *P*-value was <0.05.

## Results

### Vitamin C Does Not Affect Bacterial Growth in a Liquid Medium, but Inhibits Biofilm Formation

**FIGURE 1:**
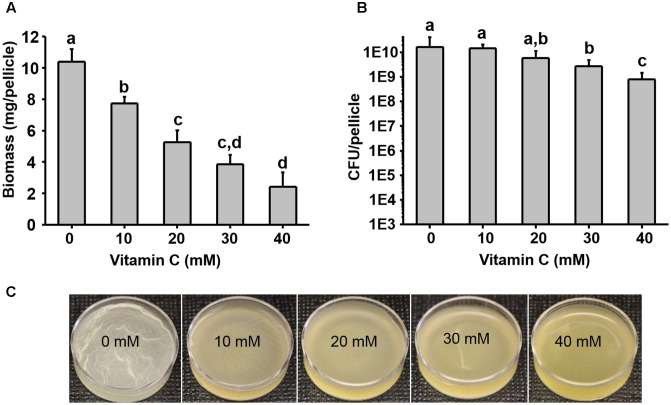
Effect of vitamin C on biomass and bacterial viability of *Bacillus subtilis* biofilm. **(A)** Biomass of a 24 h old *B. subtilis* biofilm in the presence of increasing concentrations of vitamin C. **(B)** Viability of bacteria in a 24 h old biofilm grown in the presence of increasing concentrations of vitamin C. **(C)** Representative images of a 24 h old *B. subtilis* biofilm grown with different concentrations of vitamin C. All data in **(A,B)** are mean values ± standard deviation from three biological replicates. Values labeled by the same superscript are not significantly different from each other (*P* > 0.05).

Five–ten millimeter doses of vitamin C were previously reported to completely exterminate mycobacteria (Vilcheze et al., 2013). By contrast, it has been reported that vitamin C is not bactericidal toward opportunistic pathogens such as *E. coli* and *P. aeruginosa*, but it renders them more susceptible to antibiotics and some physical treatments (Kallio et al., 2012; Khameneh et al., 2016; Helgadóttir et al., 2017). To clarify this effect of vitamin C on non-mycobacterial species, we used the opportunistic pathogens *E. coli* and *P. aeruginosa*. Since we previously hypothesized that the synergistic effects of vitamin C may be related to biofilms (Helgadóttir et al., 2017), we included also *B. subtilis*, the model organism for biofilm development (Vlamakis et al., 2013). We exposed these bacterial strains to a concentration range of vitamin C from 10 to 40 mM (sodium ascorbate, neutral pH form), assessing both the survival and growth in liquid media and biofilms. Up to 40 mM vitamin C did not significantly inhibit the planktonic growth of *B. subtilis*, *E. coli*, or *P. aeruginosa* (**Supplementary Figure S1**). However, in the same concentration range, biofilm formation was impaired for all three species (**Figure 1** and **Supplementary Figure S2**). Since the vitamin C effect was most pronounced on biofilms, we focused on *B. subtilis* for an in-depth study of the mechanism behind this effect. *B. subtilis* biofilm is the most robust and easiest to analyze in terms of structure and composition, and the mechanisms leading to its formation are well characterized (Vlamakis et al., 2013). The normal *B. subtilis* pellicle (control, 0 mM) exhibits wrinkled and folded architecture (**Figure 1C**). Vitamin C treatment abolished this wrinkled architecture and visibly attenuated the pellicle in a concentration-dependent manner (**Figure 1C**). This effect was accompanied by a linear decrease in biofilm biomass (**Figure 1A**). However, the viability of *B. subtilis* in the pellicle was not significantly affected, suggesting that the reduced biofilm biomass could be due to loss of EPS and not the loss of cells.

### Low Concentrations of Vitamin C Do Not Kill *B. subtilis* but Deplete the EPS Matrix

**FIGURE 2:**
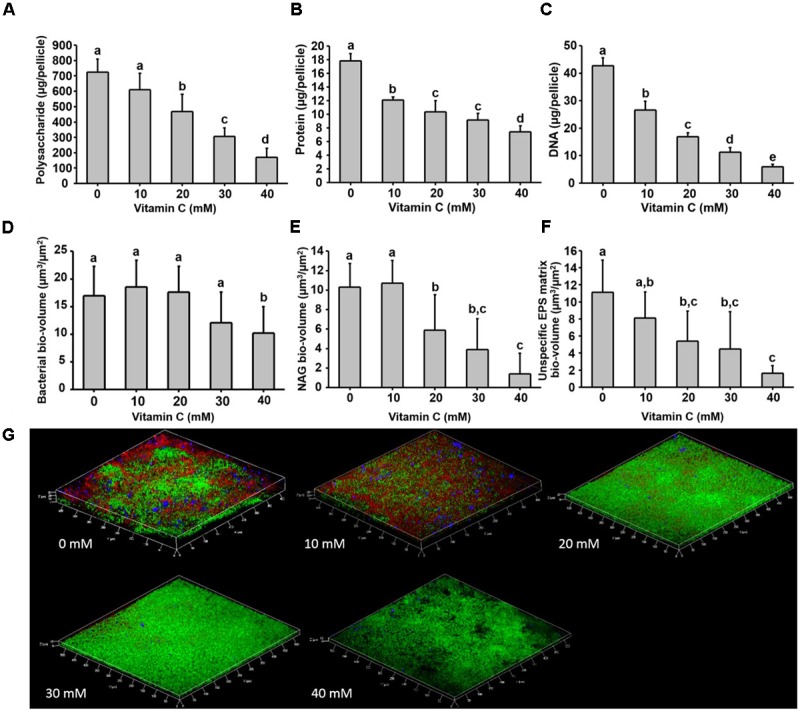
Biochemical and confocal laser scanning microscopy analysis of *B. subtilis* biofilm grown in the presence of vitamin C. **(A)** Content of polysaccharide, **(B)** content of protein and **(C)** eDNA concentration in a 24 h old biofilm. **(D)** Bacterial biovolume, **(E)** biovolume of poly *n*-acetylglucosamine and **(F)** biovolume of unspecific EPS matrix (proteins and polysaccharides) in a 24 h biofilm. **(G)** Representative 3-D architecture of a 24 h old *B. subtilis* biofilm grown in the presence of vitamin C (green: bacteria; red: *n*-acetylglucosamine; blue: unspecific EPS matrix). All data **(A–F)** are mean values ± standard deviation from three biological replicates. Values marked by the same superscripts in panels **(A–F)** are not significantly different from each other (*P* > 0.05).

**FIGURE 3:**
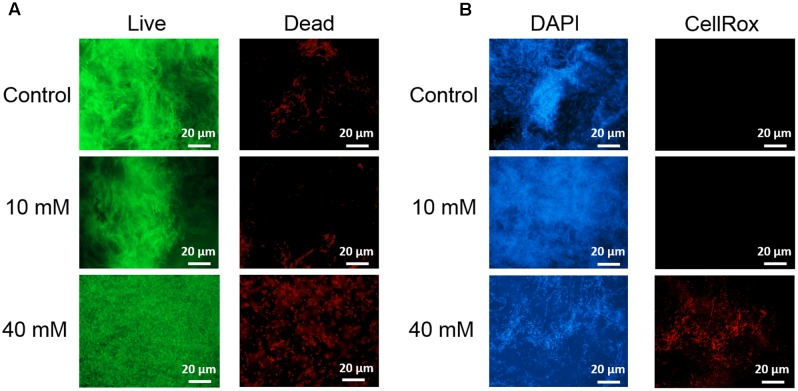
Live/dead staining of *B. subtilis* biofilm grown with or without presence of vitamin C (concentration indicated besides each image) **(A)**. Live bacteria are stained green and red bacteria are dead. Effect of vitamin C on the ROS generation in *B. subtilis* biofilm cells **(B)**. Blue color represents the bacterial cells (DAPI, nucleic acid stain), and the red color (CellRox) intensity corresponds to the amount of ROS induced in bacterial cells.

Next, we examined in details the content of various biofilm components in response to the same concentration range of vitamin C. All three major components of the biofilm matrix, polysaccharides, proteins and eDNA, were reduced in the presence of vitamin C (**Figures 2A–C**). The reduction in protein content vs. vitamin C concentration showed a linear regression coefficient of only 0.88, but the trend of exopolysaccharide and DNA reduction was more closely correlated to increasing concentration of vitamin C and the loss of pellicle biomass (**Supplementary Figure S3**). The polysaccharide content and bacterial bio-volume in the *B. subtilis* biofilm were examined by laser scanning confocal microscopy, in the same concentration range of vitamin C (**Figures 2E–G**). EPS account for over 40% of the mass of the *B. subtilis* biofilm, but their identification and characterization is not complete. Roux et al. (2015) identified that poly *n*-acetylglucosamine is the major polysaccharide component of the *B. subtilis* biofilm matrix (Roux et al., 2015). We therefore used fluorescence probes to visualize EPS components: Alexa flour, WGA conjugate, for *n*-acetylglucosamine and ConA, Tetramethylrhodamine conjugate, as an unspecific EPS binder for proteins and other polysaccharides. We first established that these probes had no effect on biofilm formation and exhibited no toxicity to *B. subtilis* cells (**Supplementary Figure S4**). The n-acetylglucosamine (stained in red) occupied a significant part of the bio-volume in the untreated sample (**Figure 2G**). With increasing concentrations of vitamin C, the overall thickness of the biofilm decreased, and the content of the poly *n*-acetylglucosamine (red) and the unspecific EPS matrix (blue) decreased as well (**Figure 2G**). The decreasing pattern of NAG with vitamin C treatment was consistent with the total polysaccharides content observed by colorimetric assay, where significant inhibition was observed with ≥20 mM of concentration. Meanwhile, the bacterial bio-volume remained constant up to 30 mM vitamin C. The decreasing pattern of bacterial bio-volume was not consistent with other results because vitamin C treatment is mainly affecting the ECM production but not the growth and viability of bacteria at lower concentrations as shown in **Figure 1**. Viability of cells was examined by live/dead staining (**Figure 3A**) where it was observed that, at low concentration of vitamin C (10 mM) the cell survival rate was similar to that of untreated control biofilms. By contrast, a significant number of cells were dead in biofilms treated with a higher concentration of vitamin C (40 mM). The killing of cells at the higher concentration of vitamin C coincided with higher levels of detectable oxidative stress (**Figure 3B**). This confirmed that vitamin C effect on biofilms takes place in two stages: at concentrations of up to 30 mM the cell viability is preserved, but there is a loss of the EPS, primarily exopolysaccharides. Above 30 mM vitamin C, the bacterial cells start dying.

### Stage 1: Low Concentrations of Vitamin C Inhibit Exopolysaccharide Synthesis, Stage 2: High Concentrations of Vitamin C Induce Lethal Oxidative Stress

**FIGURE 4:**
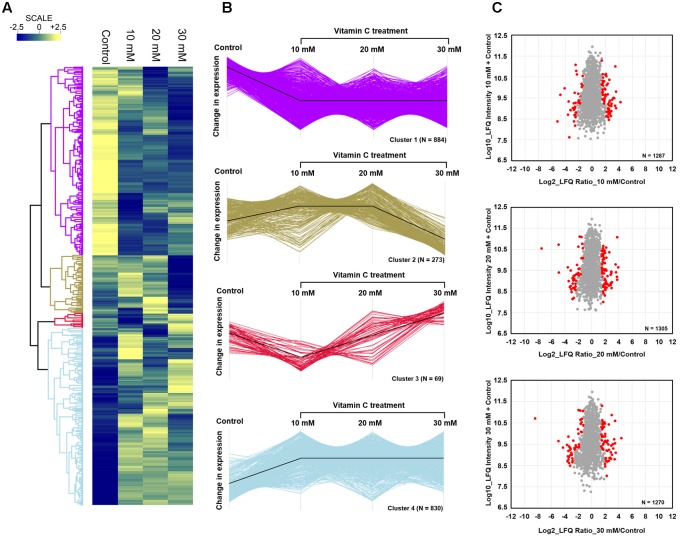
Quantitative proteome analysis of the *B. subtilis* response to vitamin C. **(A)** Hierarchical clustering analysis depicting protein clusters showing differential fluctuations. Mean LFQ intensities of the three biological replicates for each condition were calculated for the clustering analysis. **(B)** All proteins with expression change due to vitamin C grouped into four clusters with different response profiles. The black line indicates the overall trend of the protein expression profile in each of the individual clusters. **(C)** Scatter plot depicting significantly (*p* ≤ 0.05) upregulated or downregulated proteins (in red) in each of the vitamin C treatment conditions relative to the control. Log2 calculated LFQ ratios are plotted against log10 LFQ intensities.

**FIGURE 5:**
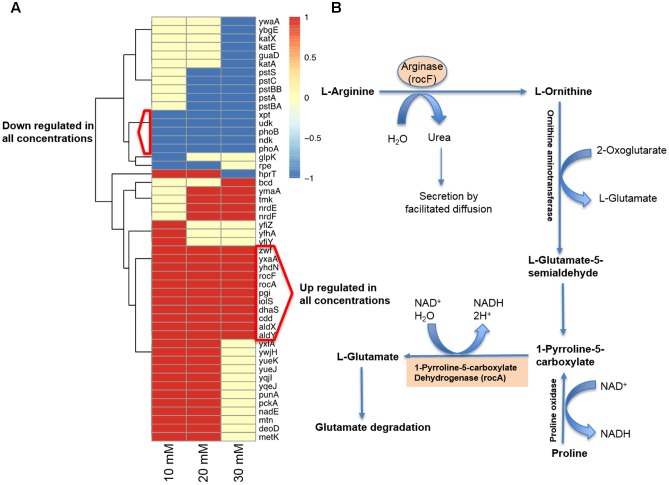
Model-guided contextualization of proteomics data. **(A)** Proteins that are associated to reactions that are significantly altered when adding increasing concentrations of sodium ascorbate to *B. subtilis*. **(B)** Degradative pathway of arginine generating P5CDH/RocA and ARGN/RocF are significantly upregulated at all concentrations of vitamin C, producing glutamate and NADH, and contributing to protection against oxidative stress.

Since vitamin C seemed to inhibit *B. subtilis* biofilm formation in two stages: reduction of EPS components at up to 30 mM (**Figure 2**), and killing of cells at 30 mM and higher by inducing the oxidative stress (**Figures 1B**, **3**), we performed an in-depth label-free quantitative proteome analysis of vitamin-C treated biofilms in this critical concentration range. A total of 2056 *B. subtilis* proteins were identified, of which 1373 were quantified (**Supplementary Table S1**). Three biological replicates showed a high degree of correlation (Pearson correlation coefficient ≥ 0.9) (**Supplementary Figure S5**). Hierarchical clustering analysis was employed for grouping similar expression profiles of proteins (**Figures 4A–C**). Differentially regulated proteins grouped in four clusters (**Figure 4B**). The majority grouped in clusters 1 (expression reduced upon the addition of vitamin C) and 4 (expression enhanced upon the addition of vitamin C). Clusters 2 and 3 showed variation across the range of vitamin C concentrations. All proteins falling in the four different clusters are listed in the **Supplementary Table S1**. Proteins for which the expression levels were most strongly affected by vitamin C treatment (*p* ≤ 0.05) were identified by plotting log2 transformed label-free quantification (LFQ) ratios against log10 transformed LFQ intensities (**Figure 4C**). Around 100 proteins were found in this category. Among these, at lower vitamin C concentrations, many proteins directly involved in exopolysaccharides synthesis, export and biofilm formation were depleted: notably PtkA, SlrR, SpeA, EpsC, EpsD, EpsE, EpsH, EpsI, EpsO, TuaD, Ugd (Kobayashi, 2008; Gerwig et al., 2014; Mijakovic and Deutscher, 2015). This correlated with disrupted expression of the key regulators controlling synthesis or activity of these proteins, such as ComA, RepC, Spo0A, YmdB, KinC, FloT, and PtkA (Lazazzera et al., 1999; Diethmaier et al., 2011; Yepes et al., 2012). By contrast, at higher vitamin C concentrations, oxidative stress associated proteins (Antelmann et al., 2000; Grimaud et al., 2001; Towe et al., 2007) were strongly overexpressed: MsrA, MsrB, MhqA, MhqD, AzoR2, and RocA. Individual roles of these proteins in biofilm production and oxidative stress response are reviewed in detail in the discussion section. It was evident from this dataset that vitamin C treatment provoked a two-stage global rearrangement of the cellular proteome, which correlated well to our previous observations on EPS content and cell viability. At low concentrations of vitamin C, biosynthetic pathways leading to exopolysaccharide synthesis and export were down-regulated, explaining the observed depletion of biofilm EPS. At higher vitamin C concentrations, the cell started expressing proteins to cope with excessive oxidative stress, which correlates to cell death and loss of the bacterial bio-volume in the biofilm. To assess the specific impact of this global proteome adaptation on redistribution of metabolic fluxes, we used the quantitative proteomics data to generate vitamin C concentration-specific genome-scale metabolic models, by mapping the protein intensities to the available *B. subtilis* model Bs-iYO844 (Oh et al., 2007) (**Supplementary Figure S6**). This enabled us to identify several metabolic pathways with flux redistribution provoked by vitamin C, which corroborate our findings (**Figure 5**). Notably, the model guided analysis showed that pyrroline-5-carboxylate dehydrogenase (P5CDH)/RocA had a significantly upregulated flux in the presence of vitamin C (**Figure 5**), which indicates that the cells are trying to neutralize ROS.

## Discussion

The interest toward bacterial biofilms is driven by the protection that their complex architecture offers toward antimicrobial agents (Donlan, 2002; Peterson et al., 2015). The key element of this architecture are the EPS (Romero et al., 2010; Xiao et al., 2012). Although different ratios of polysaccharides, proteins and eDNA components were reported in biofilms of different species, they collectively act as a backbone for the structural integrity and protection of the bacterial communities (Jennings et al., 2015; Klein et al., 2015; Voberkova et al., 2016). Polysaccharides and proteins of the biofilm matrix form a hydrophobic coating, which retards the penetration of antimicrobial agents and confers biofilm resistance (Epstein et al., 2011; Tiwari et al., 2017). Inhibiting EPS production is a viable strategy for fighting bacterial pathogens. Our results indicate that vitamin C, at concentrations up to 20 mM can be used to effectively disrupt bacterial biofilm formation by inhibiting EPS production.

At sub-lethal doses of vitamin C, i.e., below 30 mM, our proteomics data suggested that quorum sensing of *B. subtilis* was impaired, which is in accord with previous observations in *P. aeruginosa* (El-Mowafy et al., 2014). The major *B. subtilis* quorum sensing associated protein, the response regulator ComA, became less abundant upon vitamin C treatment. ComA affects the transcription of more than 10% of the *B. subtilis* genome, and it activates the transcription of genes for biofilm formation (Mielich-Süss and Lopez, 2015). In addition to ComA depletion, vitamin C provoked overexpression of RapC, a negative regulator of ComA activity (Lazazzera et al., 1999). Consequently, vitamin C provoked a drop in expression levels of a number of ComA-RapC-dependent proteins essential for biofilm formation, such as Spo0A, SlrR, YmdB, KinC, FloT, and SpeA. Inactivation of Spo0A, a major early sporulation transcriptional factor, causes a defect in biofilm formation due to its role in negatively regulating AbrB (Hamon and Lazazzera, 2001) and controlling the expression of an operon responsible for the synthesis of the exopolysaccharide matrix (McLoon et al., 2011). Cells with defective FloT are known to reduce the level of FtsH protease which indirectly regulates the phosphorylation and activity of Spo0A via phosphatase degradation (Yepes et al., 2012). SlrR acts in concert with SinR, and induces the *eps* and *yqxM* operons required for biofilm formation, by consequence, a mutation of *slrR* leads to a defect in biofilm formation (Kobayashi, 2008). Inactivating *ymdB* has been demonstrated to suppress SinR-dependent biofilm gene expression (*slrR*, *tapA*, *epsA*) and to induce the expression of SigD dependent motility genes (*hag*, *cheV*, and *motA*) (Diethmaier et al., 2011). SpeA, an arginine decarboxylase, is essential for the production of polyamines which are required for *B. subtilis* biofilm formation (Burrell et al., 2010). Finally, low concentrations of vitamin C inhibited the expression of a number of proteins involved directly in synthesis and export of extracellular polysaccharides (Cluster 1), namely the operon *epsA-O* (Barnes et al., 2012; Pozsgai et al., 2012). Among the *eps* genes, *epsH-K* encodes proteins responsible for the production of poly-n-acetyl glucosamine (Roux et al., 2015), *epsH*-*J* encodes glycosyltransferases, while *epsK* is an exporter of poly-*N*-acetylglucosamine. EpsE has been demonstrated to have a dual function: production of exopolysaccharides and functional control of the flagellum (Guttenplan et al., 2010). In our dataset, proteins EpsC, EpsD, EpsE, EpsH, EpsI, and EpsO were no longer detectable in the presence of vitamin C. Similarly, UDP-glucose dehydrogenases TuaD and Ugd, which synthesize glucuronic acid, a precursor for exopolysaccharide production (Mijakovic and Deutscher, 2015), were also less abundant in vitamin C-treated samples. The activity of several key proteins in the exopolysaccharide production and export cluster are known to be positively regulated by tyrosine-phosphorylation, catalyzed by BY-kinases (Mijakovic et al., 2003; Whitfield and Larue, 2008). In our dataset, the expression BY-kinase PtkA, a control protein for exopolysaccharide production and biofilm formation, was also strongly repressed in the presence of vitamin C. While we clearly observe the consequences of disruption of specific regulatory networks that control the EPS synthesis on the proteome level, it is still unclear how vitamin C targets these regulators. Further studies will be needed to elucidate the exact molecular mechanism behind this effect.

The killing effect of vitamin C against *B. subtilis* biofilm cells occurred at concentrations of 30 mM and above (**Figures 1A**, **2D**). It has been previously demonstrated that the bactericidal effect of vitamin C against mycobacteria was mainly associated with oxidative stress (Roux et al., 2015). Accordingly, in our dataset several proteins known to provide protection against oxidative stress (Towe et al., 2007) were overexpressed at high vitamin C concentrations: MsrA, MsrB, MhqA, MhqD, and AzoR2 (**Figure 4B**, cluster 5). MsrA and MsrB belong to the methionine sulfoxide reductase (Msrs) family and are known to protect cells from oxidative stress by reducing methionine sulfoxide to methionine (Grimaud et al., 2001). Both MsrA and MsrB mutants of *E. coli* were shown to have more sensitivity toward oxidative stress generated by H_2_O_2_ (Towe et al., 2007). It has been reported that genes belonging to the MhqR regulon: *mhqA*, *mhqD*, and *azoR2* are overexpressed under the electrophile and oxidative stress (Antelmann et al., 2000). The glyoxalases (MhqA, MhqE, and MhqN) were also demonstrated as critical for the detoxification of cytotoxic methylglyoxal in bacteria and eukaryal cells (Antelmann et al., 2000). Azoreductases (AzoR1/2) are enzymes which catalyze the NADH dependent two-electron of substrates to protect the cells from toxic effects of free radicals and ROS arising from one-electron reduction (Antelmann et al., 2000). In addition, pyrroline-5-carboxylate dehydrogenase (P5CDH)/RocA was overexpressed, leading to a significantly higher predicted flux rate in vitamin C-treated genome-scale metabolic models (**Figure 5B**). P5CDH converts Δ^1^-pyrroline-5-carboxylate (P5C) to glutamate. Proline oxidase (YcgM), which degrades proline into P5C, was not upregulated in the presence of vitamin C. By contrast, arginase (ARGN/RocF) had a significantly higher flux in all vitamin C models, as well as the secretion of urea, a byproduct of the arginase reaction. Accumulation of P5C was found to induce cell death by producing reactive oxygen species (Nishimura et al., 2012), and the activation of P5CDH counters that effect (Miller et al., 2009). Therefore, it is plausible that vitamin C-induced oxidative stress provokes the overexpression of RocA, as a protective effect. We propose that this oxidative stress, clearly evidenced by the proteome rearrangement, is the most probable cause of death for cells that become exposed once the protective EPS matrix is lost, i.e., at elevated concentrations of vitamin C, above 30 mM.

Based on these findings, we propose that the inhibitory effect of vitamin C on biofilm formation proceeds by inhibition of quorum sensing and other stationary phase regulatory mechanisms underpinning biofilm development, which specifically leads to inhibition of polysaccharide biosynthesis. Once the EPS content is reduced, at vitamin C concentrations of 30 mM and above in the case of *B. subtilis*, bacterial cells get fully exposed to the medium. Thereby they become more susceptible to killing by vitamin C-induced oxidative stress reported here, and other antibacterial compounds or treatments (Khameneh et al., 2016; Helgadóttir et al., 2017). In situations where the risk of developing resistance by administering excessive doses of antibiotics is too high, we would argue that low concentrations of vitamin C can be effectively used as a pre-treatment or a combined treatment to destabilize bacterial biofilms.

## Author Contributions

SP, VR, AA-H, AD, and CS performed the experiments. SP, VR, AD, AA-H, VM, KM, TG, XG, FW, and IM analyzed the data. SP, VR, AA-H, and IM wrote the manuscript with support from all authors.

## Conflict of Interest Statement

The authors declare that the research was conducted in the absence of any commercial or financial relationships that could be construed as a potential conflict of interest.

## Supplementary Material

The mass spectrometry proteomics data have been deposited to the ProteomeXchange Consortium via the PRIDE (Vizcaíno et al., 2013) partner repository with the dataset identifier PXD007533.

FIGURE S1 Effect of vitamin C on bacterial growth. Growth of **(A)** *Bacillus subtilis*, **(B)** *Escherichia Coli*, and **(C)** *Pseudomonas aeruginosa*, in the presence of different concentrations of vitamin C, as indicated in the color-coded legend. The experiment was performed with biological triplicates, and the error bar shows the standard deviation.

FIGURE S2 Effect of vitamin C treatment on biofilm formation by *E. coli* and *P. aeruginosa*. The biofilms were stained with crystal violet and optical density was measured. All data represent mean ± standard deviation. Values followed by the same superscripts are not significantly different from each other (*P* > 0.05).

FIGURE S3 Relationship between concentration of sodium ascorbate and biomass and constituents of EPS matrix. Linear fitting of biomass, polysaccharide, DNA and protein of biofilm vs. increasing concentration of vitamin C.

FIGURE S4 Effect of polysaccharide fluorescence stain on biofilms. Biomass **(A)** and viability **(B)** of *B. subtilis* biofilm grown in the presence of two different polysaccharide stains alone or in combination: Concanavalin A Tetramethylrhodamine conjugate (ConA; 50 μg/ml) and Alexa flour^®^ 633-labeled wheat germ agglutinin conjugate (WGA; 10 μg/ml).

FIGURE S5 Correlation between biological replicates of the proteomics analysis. Correlation plots depicting correlation of proteins between biological replicates in the control and in 10, 20, and 30 mM vitamin C treatment conditions. The figure shows Log10 intensities of individual replicates plotted against each other.

FIGURE S6 Workflow for model-guided analysis of proteomics data. **(A)** Log10 LFQ protein intensities from the biological replicates were averaged and mapped using GIMME (Becker and Palsson, 2008) to Bs-iYO844, the *B. subtilis* genome-scale metabolic model (Oh et al., 2007) to constrain the fluxes in the associated reactions. **(B)** The range and distribution of feasible metabolic flux for each reaction were determined by using Markov Chain Monte Carlo (MCMC) sampling (Lewis et al., 2012). Averaged sampled predicted flux distributions for each reaction at each sodium ascorbate concentration were compared to those from the control model (ascorbic acid concentration = 0 mM) in order to identify differentially active reactions which are later decomposed into their corresponding genes through the gene-protein reaction associations embedded in the model.

TABLE S1 List of all identified proteins, proteins belonging to cluster 1, 2, 3, 4 from **Figure 3**, and regulated proteins.
