# Supplementary figures and images for: Low Concentrations of Vitamin C Reduce the Synthesis of Extracellular Polymers and Destabilize Bacterial Biofilms
Source: Front Microbiol. 2017 Dec 22;8:2599. doi: 10.3389/fmicb.2017.02599 (PMC5748153; doi:10.3389/fmicb.2017.02599)

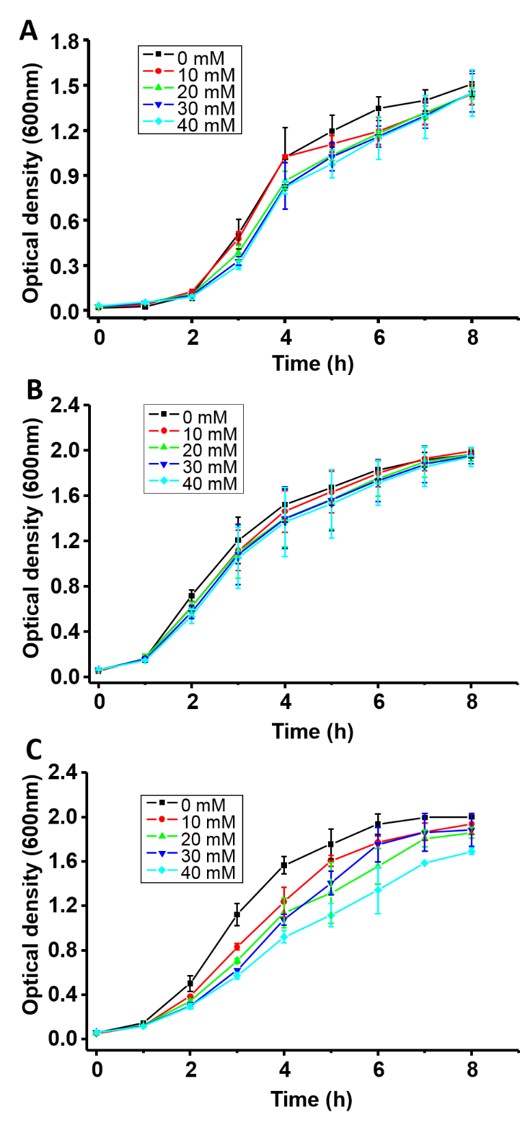

Supplement: FIGURE S1 — Effect of vitamin C on bacterial growth. Growth of (A) Bacillus subtilis, (B) Escherichia Coli, and (C) Pseudomonas aeruginosa, in the presence of different concentrations of vitamin C, as indicated in the color-coded legend. The experiment was performed with biological triplicates, and the error bar shows the standard deviation. [file Image_1.JPEG]

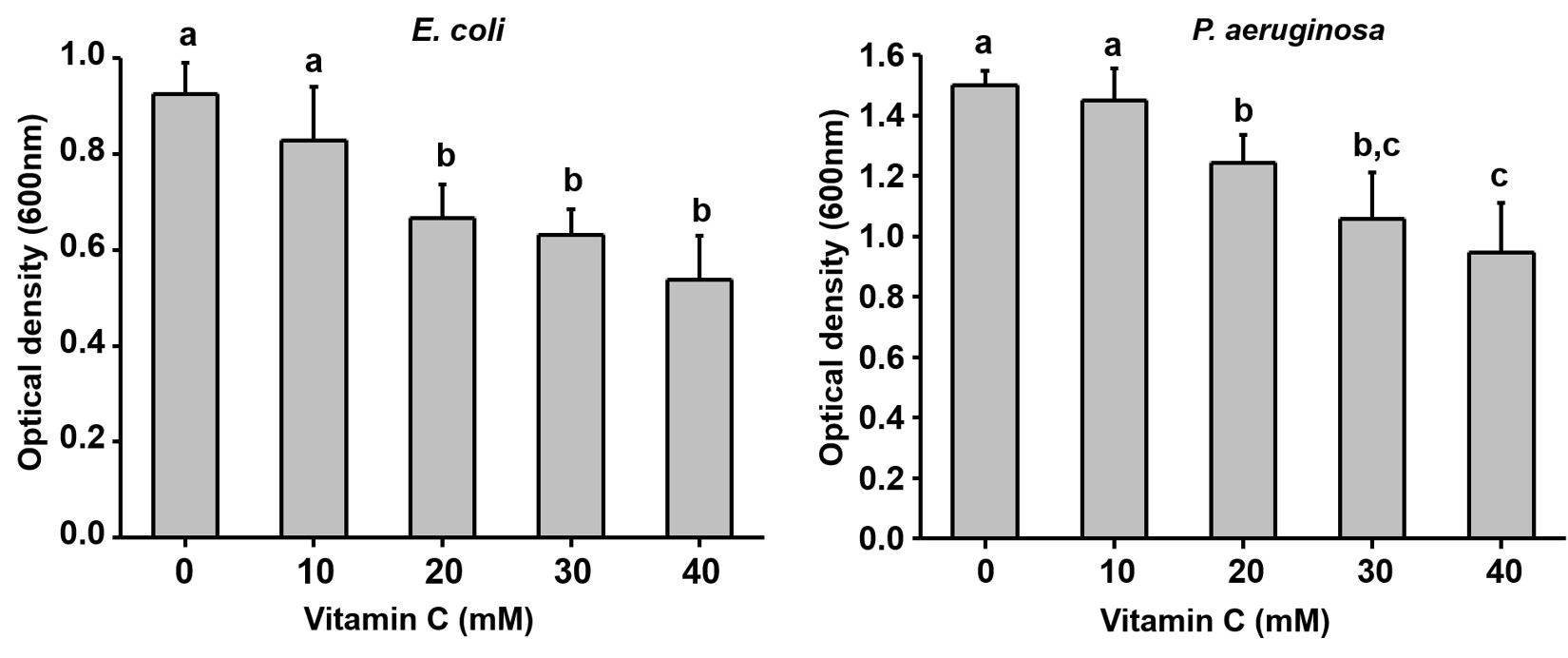

Supplement: FIGURE S2 — Effect of vitamin C treatment on biofilm formation by E. coli and P. aeruginosa. The biofilms were stained with crystal violet and optical density was measured. All data represent mean ± standard deviation. Values followed by the same superscripts are not significantly different from each other (P > 0.05). [file Image_2.JPEG]

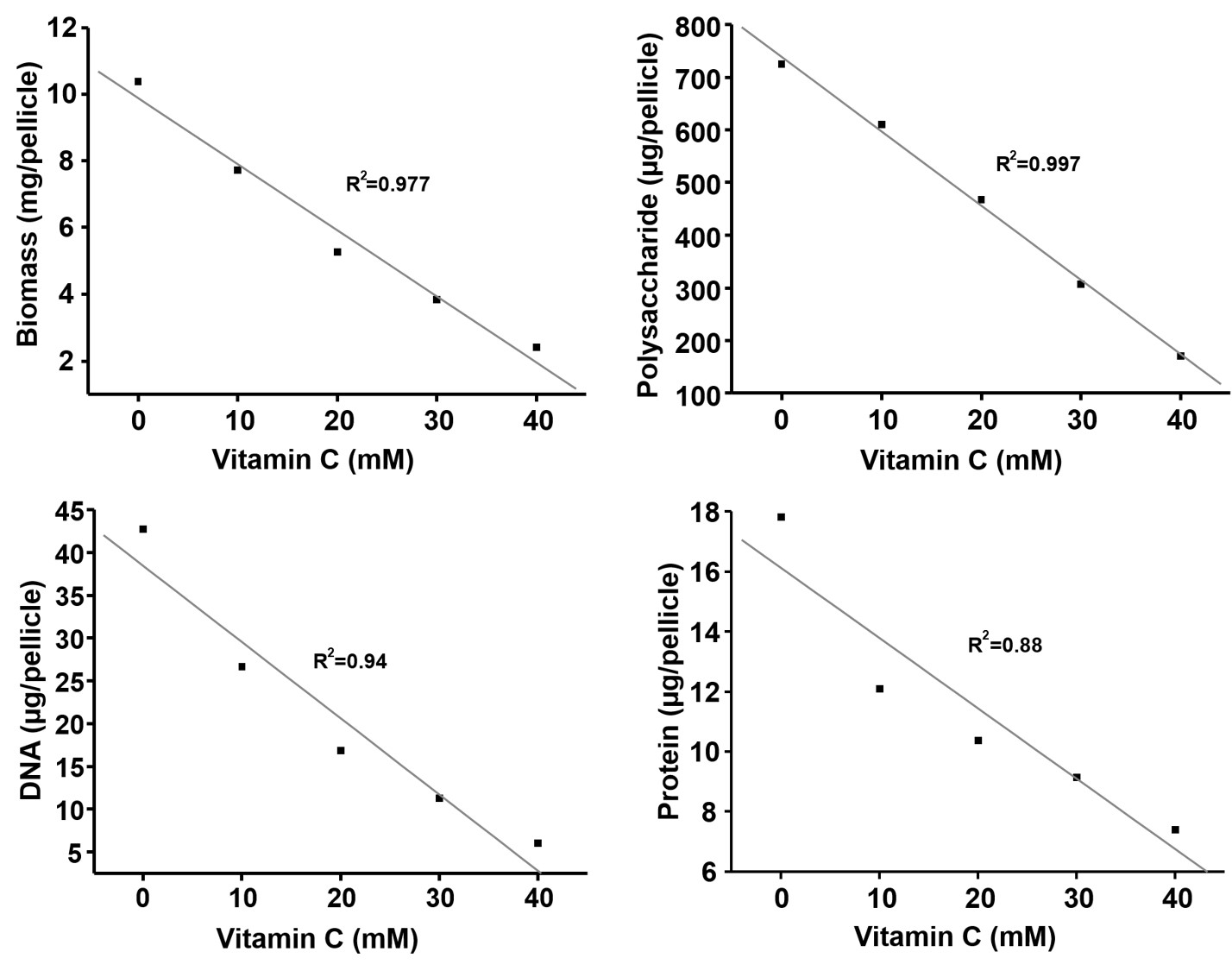

Supplement: FIGURE S3 — Relationship between concentration of sodium ascorbate and biomass and constituents of EPS matrix. Linear fitting of biomass, polysaccharide, DNA and protein of biofilm vs. increasing concentration of vitamin C. [file Image_3.JPEG]

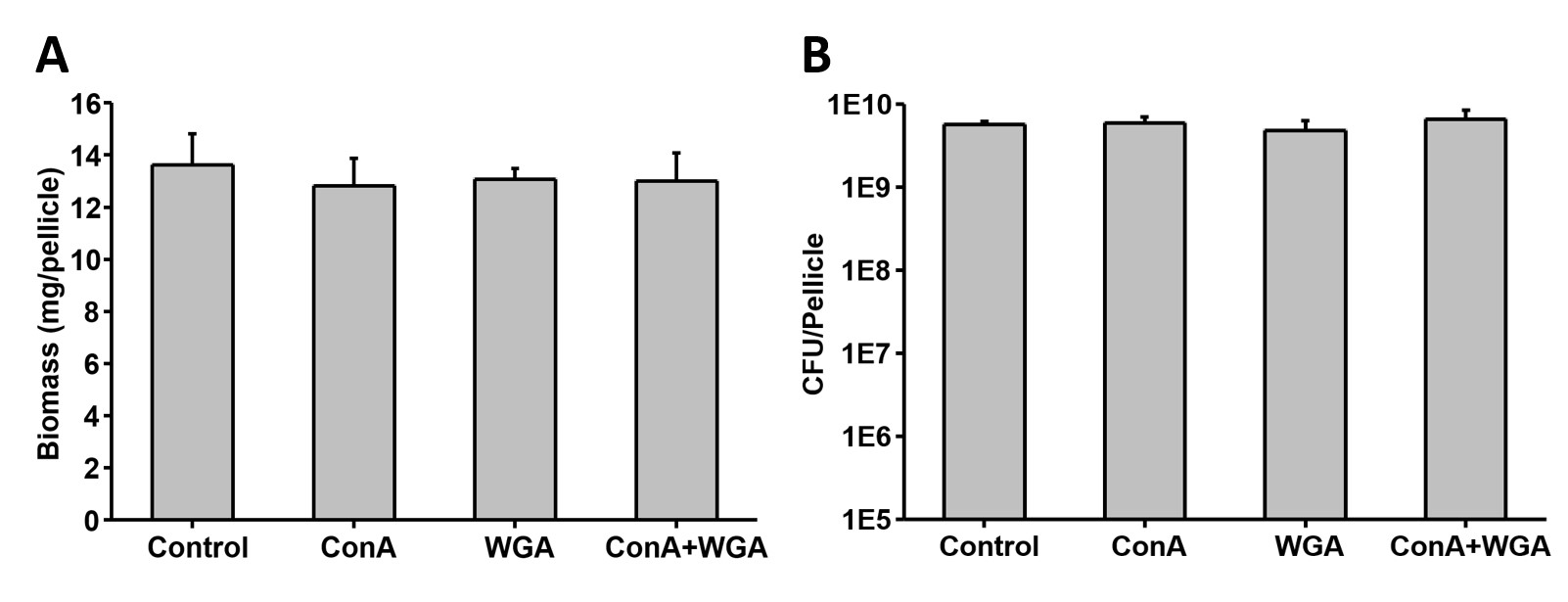

Supplement: FIGURE S4 — Effect of polysaccharide fluorescence stain on biofilms. Biomass (A) and viability (B) of B. subtilis biofilm grown in the presence of two different polysaccharide stains alone or in combination: Concanavalin A Tetramethylrhodamine conjugate (ConA; 50 μg/ml) and Alexa flour® 633-labeled wheat germ agglutinin conjugate (WGA; 10 μg/ml). [file Image_4.JPEG]

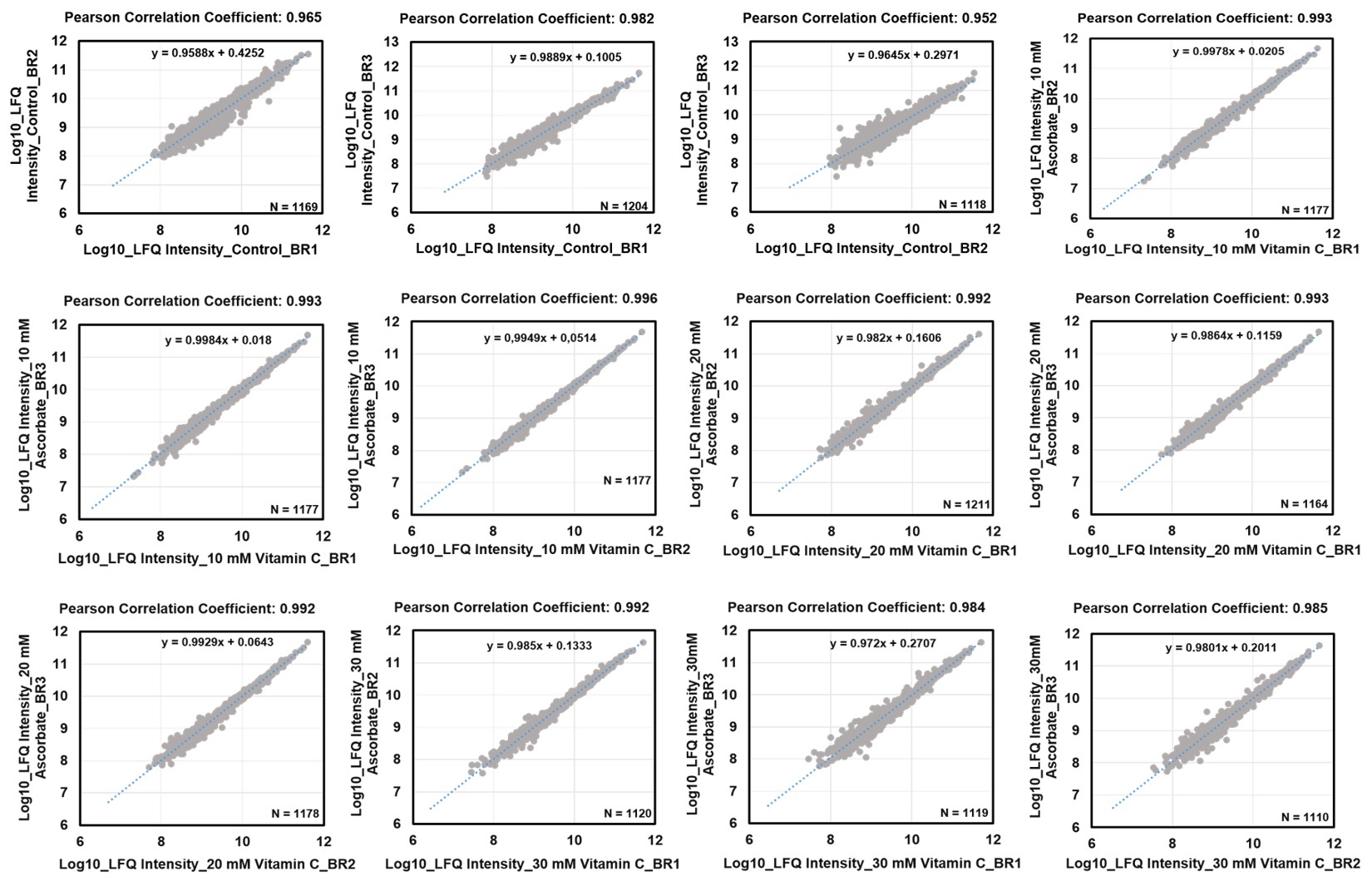

Supplement: FIGURE S5 — Correlation between biological replicates of the proteomics analysis. Correlation plots depicting correlation of proteins between biological replicates in the control and in 10, 20, and 30 mM vitamin C treatment conditions. The figure shows Log10 intensities of individual replicates plotted against each other. [file Image_5.JPEG]

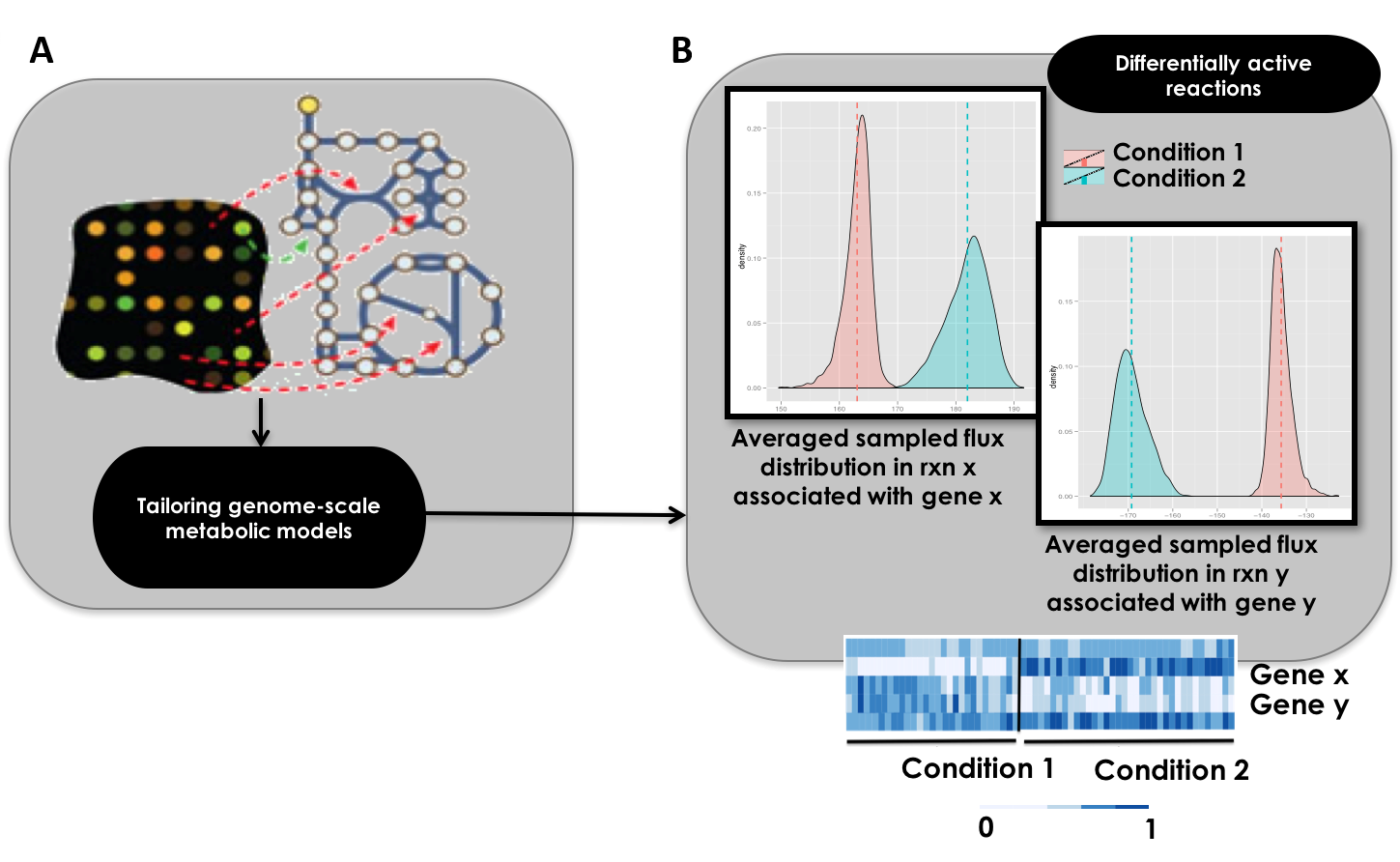

Supplement: FIGURE S6 — Workflow for model-guided analysis of proteomics data. (A) Log10 LFQ protein intensities from the biological replicates were averaged and mapped using GIMME (Becker and Palsson, 2008) to Bs-iYO844, the B. subtilis genome-scale metabolic model (Oh et al., 2007) to constrain the fluxes in the associated reactions. (B) The range and distribution of feasible metabolic flux for each reaction were determined by using Markov Chain Monte Carlo (MCMC) sampling (Lewis et al., 2012). Averaged sampled predicted flux distributions for each reaction at each sodium ascorbate concentration were compared to those from the control model (ascorbic acid concentration = 0 mM) in order to identify differentially active reactions which are later decomposed into their corresponding genes through the gene-protein reaction associations embedded in the model. [file Image_6.TIF]
